# A Matching Study on the Influence of Advertised Information Expression and Product Type on Consumer Purchase Intention

**DOI:** 10.3389/fpsyg.2022.859959

**Published:** 2022-04-01

**Authors:** Qiang Yang, Shanshan Liu, Yao Li, Haifeng Kang

**Affiliations:** School of Management, Tianjin University of Technology, Tianjin, China

**Keywords:** direct expression advertising, metaphorical advertising, product type, information-processing fluency, personal involvement, purchase intention

## Abstract

Due to extensive product differentiation and the personalized aesthetic needs of consumers, modern enterprises need different expressions of information to attract consumers’ interest and improve their purchase intention. This study draws from the elaboration likelihood model, anchoring theory, and media richness theory to explore how the expression of advertised information can be effectively matched to the product type to enhance consumers’ purchase intention. The mediating effect of information-processing fluency and moderating effect of consumers’ personal involvement on this relationship is also explored. Data from experiments and questionnaires involving 1,292 participants were analyzed. The results show that direct expression of advertised information is more suitable for advertising search products, while metaphorical expressions of advertised information are more suitable for advertising experience products. These combinations of product type and expression of advertised information can effectively improve consumers’ purchase intention. Meanwhile, the main combined effect of the product type and expression of advertised information is mediated by consumers’ information-processing fluency, and moderated by consumers’ personal involvement positively.

## Introduction

Advances in technology and the emergence of mobile Internet communication channels have made available an increasing variety of product types. As a consequence, today’s consumers face increasing choices between products that are advertised with different expressions of information (Urbinati et al., 2019). In the past, information in advertisements was often expressed in a direct expression manner, which has been attracting more attention by scholars (Verhagen and Bloemers, 2018; Kim et al., 2019). However, such an advertising strategy was unsuitable for publicizing some types of products (e.g., hotel service and facial masks) and failed to meet the aesthetic needs of diversified consumption. In response to these shortcomings of the direct expression advertising strategy, strategies using the metaphorical expression of advertised information have been developed, which more focuses on the use of products and elicit in the minds of audiences (Chang and Yen, 2013; Mohanty and Ratneshwar, 2015; Lee et al., 2019).

Advertising serves as an important carrier of information from enterprises to consumers. Through advertising, product information can be effectively communicated to consumers. Based on this information, consumers form perceptions of the products and respond accordingly. Therefore, how advertised information is expressed will affect consumers’ attitudes toward a product, thereby affecting their purchase intention (Wang et al., 2019). Products will be differentiated according to their characteristics, such as forms, durability (Chang et al., 2018), the involvement level (Verhagen and Bloemers, 2018), and so on. Thus, it is necessary to match the expression of advertised information with different types of products to promote positive perceptions of the products among consumers. The processes determining how products are purchased may involve different mechanisms of social influence, such as cognition process or psychological stimulation (Iyengar et al., 2015). The researches also suggest that metaphorical advertising can enhance consumers’ motivation to deal with the advertised information through the inferences or meanings they elicit in the minds of audiences (Lopez-Gonzalez et al., 2018; Lee et al., 2019) and encourage consumers to develop unique inferences and positive attitudes about the brand (Wien and Peluso, 2021). Thus, whether to use metaphors in advertising will be varied in their impact on product appreciation and behavioral intention among the target audiences (Lee et al., 2019). As mentioned above, it is necessary to explore ways to effectively match specific types of products to the expression of advertised information and enhance consumers’ purchase intention. Additionally, factors, such as transmission of advertised information and consumers’ subjective perceptions of the complexity of advertised information, can influence the effectiveness of advertising campaigns (Verhagen and Bloemers, 2018; Chen et al., 2021) and thus further shape consumers’ attitudes toward the product. From the perspective of information processing, this study investigates the mediating path of the main combined effect of the product type and expression of advertised information on consumers’ purchase intention. Thus, the study comprehensively reveals the direct and indirect combined effects of the expression of advertised information and product type on consumer purchase intention.

In addition, the research suggests that the crowded social environment leads consumers to browse advertised information quickly and decreases their willingness to expend additional energy to undertake in-depth analyses of the advertised information; instead, they rely on information that is concise and clear. While, the research also suggests that a consumer with a busy lifestyle is attracted by interesting advertised information and to remember such information; however, this form of advertising may be a potentially risky advertising model for enterprises (Cameron and Maslen, 2010). Thus, which type of advertised information expression will be better is still unknown. Our study suggests that considering contingency factors, such as individual characteristic, may help to fill the gap. Meanwhile, the abilities of consumers to comprehend advertised information may vary, as may their memory capacities and states of arousal. Furthermore, consumers’ desires for products may vary with time, causing them to hold different attitudes toward the final products. For these reasons, research on the effects of advertising on consumer purchase intention needs to consider the influence of individual characteristics. Scholars have suggested that personal involvement affects their associations with advertising and the products advertised (Leow et al., 2019), resulting in different levels of attention and investment devoted to the deconstructive process of product advertising. Personal involvement had crucial attribute for online retailing and marketing research (Leckie et al., 2016; Harmeling et al., 2017). Thus, our study further considers the contingency influence of consumers’ personal involvement on the effect of advertising.

This study makes three key contributions to the extant literature. First, previous studies mainly focus on the effects of singular expressions of advertised information (Kim et al., 2019) or product type (Iyengar et al., 2015; Cancela et al., 2021) on consumers’ purchase intention, the study draws on anchoring theory and media richness theory to develop an integrative theoretical framework, linking both the expressions of advertised information (i.e., direct expression and metaphorical expression) and product types (i.e., search product and experience product) to consumers’ purchase intention. Second, the empirical model investigates the matching effects between the expressions of advertised information and product types on consumers’ purchase intention. The findings reveal that direct expressions of information in advertising can better stimulate consumers’ purchase intention for search products than metaphorical expressions, while, for experiential products, metaphorical expressions of information in advertising can better stimulate consumers’ purchase intention. The conclusions reconcile related research arguments about the relative effectiveness of different expressions of advertised information. Third, previous studies mainly focus on the direct effects of expressions of advertised information (Kim et al., 2019), neglecting the possible mediating path or moderating effects of individual characteristics. Thus, the study further explores the mediating effect of information-processing fluency and moderating effect of personal involvement, revealing the path and mechanisms of combined effects of the expressions of advertised information and product types on consumers’ purchase intention completely. Thus, our study not only enriches conclusions but also expands the domain of advertising research. Meanwhile, the study provides theoretical insights into the effective design of advertising to enhance consumers’ purchase intention.

## Theoretical Background and Hypotheses

### Product Type

In the context of research on traditional shopping, the American Marketing Association (AMA) initially classified products into “convenience products,” “optional products,” and “special products,” based on the time and energy spent by consumers in purchasing behavior. However, products have been reclassified with the advent of the Internet. Based on the differences in how product information is captured and the degree to which consumers can understand the products, some authors have classified products as “search products” and “experience products” (Chen et al., 2021; Mirhoseini et al., 2021). In addition, products can be classified as “high involvement products” and “low involvement products” based on differences in the subjective states of arousal that they induce in consumers or the amount of attention they draw from consumers (Seyed and Maryam, 2018; Verhagen and Bloemers, 2018). Wang et al. (2022) have classified the products according to whether the sensory state is bright or not. Finally, products have also been classified as “utilitarian products” and “symbolic products” based on the differences in consumers’ functional needs related to the products (Spiller and Belogolova, 2016; Wien and Peluso, 2021). As the study investigates the effects of advertised information expressions and information-processing fluency from the lens of information, we need to consider both the characteristics of advertised information and consumers’ demands (Bi et al., 2017; Morimoto, 2017). Specifically, advertised information needs to be rapidly propagated, reach a wide range of consumers, and dynamically respond to their personalized demands (Ojansivu and Medlin, 2018). And consumers’ demands are with high level of convenience, timeliness, and comfortableness. With these in mind, we adopt the classification of products by Huang et al. (2009), distinguishing between search products and experience products. Based on the distinction between search and experience product, it helps us to measure sources of uncertainty and to what extent consumer reviews help resolve uncertainty, especially suffering from complexity in digital context. Search products refer to products for which consumers can objectively evaluate the main attributes by using the available information before making their purchase; consumers can thus decide whether or not to purchase search products through the introduction of product parameter. Often, search products are highly standardized and emphasize information features; examples include digital cameras, tablets, and smartphones. Experience products are products that are difficult to compare, consumers need to purchase and use these products before they can develop subjective feelings about them. Experience products mainly emphasize experience attributes; examples include food and beverages, cosmetics, and entertainment services (Yan et al., 2016). As mentioned above, consumers will perceive different cognition and make purchase decision accordingly (Iyengar et al., 2015). It is necessary to distinct different types of products to meet consumers’ demands.

### The Expression of Advertised Information

The content and expression of advertised information is rapidly growing, owing to progress in science and technology, as well as the evolution of marketing strategies. Enterprises can not only distribute fragments of product information over time (Cheong et al., 2021), but also use the repetition of language and imagery in the advertised information during the early stage of a campaign, discreetly and strongly stimulating consumers’ purchase motivations. Such advertising strategies can strongly improve the overall effectiveness of communication (Kim et al., 2019). In traditional advertising, information is mainly expressed in language. To avoid boring narratives and to attract and captivate consumers’ attention, traditional advertising not only pursues a practical design but also integrates imaginative and aesthetic designs into the expression of advertised information (Venkatraman et al., 2021). This achieves an aesthetic design that is sensitive to consumers’ aesthetic needs and effectively disseminates information about the product’s function, thereby triggering consumers’ purchase motivations. With the goal of effectively communicating advertised information to trigger consumers’ purchasing motivations in the new era of marketing, our study focuses on advertised information that is expressed in text and further classifies this information into different forms of expression.

In mainstream research, the different forms of expression of advertised information are mostly classified based on the perspectives of linguistics, communication, and cognitive psychology (Della Bitta et al., 1981; Fuhrich and Schmid, 2016). In line with the characteristics of China’s online shopping industry, this study classifies the expression of advertised information into two forms: direct expression and metaphorical expression (Chang et al., 2018). In this study, a direct expression is defined as an expression that is simple, natural, easy to understand, and similar to vernacular expression. A metaphorical expression is defined as an expression that is implicit, interesting, and stimulates the consumer’s imagination. The deconstruction of product information, consistency of information expression, and cognitive demands required from consumers underlined these two forms of expression are different. As such, consumers’ abilities to acquire a subjective understanding of advertised information are expected to differ between these two forms of expression (Mohanty and Ratneshwar, 2015). The literature suggests that most direct advertisements are plain, straightforward, and akin to instructions explaining how products should be used. In other words, information about product function is simply and straightforwardly displayed to consumers. In contrast, the semantic associations embedded in metaphorical advertising are implicit and can be explained in terms of cognitive, affective, and motivational processes. A consumer can only fully grasp the appeal of metaphorical advertising if he has greater mobilization of cognitive resources and greater cognitive elaboration. And then the consumer can understand the similarity between the product subject and the advertising carrier (Chang et al., 2018; Uddin, 2019).

Research has shown that direct expression advertising, which uses simple and intuitive text, has the advantage of helping consumers to understand and accept product information more easily, thereby enhancing their trust and perceived reliability of the brand (Hur et al., 2020). In comparison, metaphorical advertising that uses text can promote vitality and complex stimulation through the multi-dimensional expressions, such as the unique use of vivid ideas, color, sound, and imagery (Piekkari et al., 2020), to establish unique and persuasive concepts relating to the product (Myers and Jung, 2019). Metaphorical advertising can inject novelty into advertising and enhance consumers’ motivation to process the advertised information (Lopez-Gonzalez et al., 2018; Lee et al., 2019). These properties can further encourage consumers to develop multiple, distinct inferences and positive attitudes about the brand (Tjitrakusuma, 2016). Nonetheless, it remains unclear how different expressions of advertised information may comparatively influence the overall success of advertising.

### The Interaction Between Product Type and the Expression of Advertised Information

We suggest that the success of advertising depends on not only how the advertised information is expressed but also the product type (Iyengar et al., 2015). When designing advertisements for different types of products, enterprises strive to enhance the attractiveness of the advertisements by including elementary symbols or using unique language related to products (Zhou et al., 2010). In addition, media richness theory posits that the most well-matched combination of advertising will meet consumers’ demands more effectively than others. As information can be interpreted in a variety of ways, reducing the ambiguity of information is the key to achieve an effective transmission of information (Daft and Lengel, 1984). Thus, we hope to identify effective strategies for advertising and marketing by considering the potential matching effects of product type and the expression of advertised information.

Anchoring theory states that an anchoring point for an initial belief arises when the audience begins to observe advertisements for the product (Van Auken and Adams, 2005). Subsequently, in accordance with the elaboration likelihood model, individuals process information through the central route (CR) and peripheral route (PR). In comparison with PR, a CR is associated with a higher degree of fine processing and higher cognitive needs (Zhou et al., 2016; Allison et al., 2017). Specifically, when advertised information is presented directly to a consumer, that consumer’s psychological defenses are reduced, and they are likely to engage in rational thinking, thereby forming an anchor point for the initial belief, “this is easy to understand” (Rigolizzo, 2018). Subsequently, to avoid omissions and determine whether main information about the product is present, the consumer compares or analyzes the information at a deeper level. In contrast, product advertising that uses metaphorical expressions requires the consumer to have the ability to understand and translate the information (Olkoniemi et al., 2016). The consumer may associate the advertised information with other things that are not directly related to product information and engage emotional thinking as the primary means to form the anchor point for the initial belief, “this is interesting and worth thinking about.” In such a way, metaphorically expressed advertised information encourages consumers to be awakened to previous similar experience as the advertised experience, and it causes them to process information through the PR. Search products are characterized by standardization. After a consumer searches out the main information about a product, he or she can conduct a comparative analysis to evaluate and make decisions about the product without the need to obtain relevant information through purchase or use (Acar et al., 2021). A simple and explicit expression of information can effectively and accurately provide relevant information about search products, appeal to consumers’ rational thinking, induce cognitively oriented benefits, and improve the efficiency and effectiveness of communicating product information to consumers (Benlian et al., 2012). The direct and concise expression of information in direct expression advertising is likely to meet consumers’ demand for information about the search product. Therefore, we propose that direct expression advertising is more suitable for advertising search products.

In contrast to search products, experience products have personalized features. Such products usually need to be purchased or used before their quality can be judged; that is, decisions about experience products cannot be made through a standardized information search (Girard and Dion, 2010; Lim et al., 2015). Therefore, consumers tend to explore potential personalized information from advertising. Advertisers of experiential products often make connection with consumers through implicit and interesting advertised information further deepen consumers’ understandings of experiential products (Gallo et al., 2019). The elegant and personalized expression of information in metaphorical advertising can meet consumers’ demands for information about experience products. Therefore, we propose that metaphorical advertising is more suitable for advertising experience products. Based on the above arguments, we propose the following hypothesis:

*H1*: Direct expressions of information in advertising can better stimulate consumers’ purchase intention for search products than metaphorical expressions. In contrast, metaphorical expressions of information in advertising can better stimulate consumers’ purchase intention for experience products than direct expressions.

### Mediating Effect of Consumer Information-Processing Fluency

Consumers’ perceptions of advertised information often vary (Aguirre-Rodriguez, 2013). “Information-processing fluency” refers to the level of ease an individual experiences in acquiring a subjective understanding of the information encountered (Tsai and McGill, 2011). Information-processing fluency is an intuitive judgment—a cognitive process that occurs at the unconscious level. It affects the speed at which an individual processes information, as well as the attention to and evaluation of the processed object, further influence consumers’ perception (Nunes et al., 2015). Cue consistency theory (CCT) posits that the perception of consistent (vs. inconsistent) information better enables consumers to make effective judgments and final decisions (Nunes et al., 2015). Consumers that perceive ease during information processing are more likely to develop consistent perceptions and generate positive evaluations of the advertised information and the products advertised (Novemsky et al., 2007), thus enhancing their purchase intention.

We suggest that when search products are matched with direct expression advertising and experience products are matched with metaphorical advertising, consumers’ requirements for product information are consistent with the output of information advertised. This matching effectively promotes consumers’ subjective understanding of the advertised information, thereby raising their information-processing fluency. The study suggests that information-processing fluency has positive effect on the familiarity that the consumer perceived of the advertising stimulation (Ferraro et al., 2016), and the confidence in making decisions and choices on products (Tsai and McGill, 2011). Thus, the matching effect of product type and advertising expression positively improve consumers’ understanding of the product and brand identity. Thus, we propose the following hypothesis:

*H2*: Consumer information-processing fluency mediates the combined effect of product type and advertising expression on consumers’ purchase intention.

### Moderating Effect of Personal Involvement

Involvement was concerned in psychological domain originally, which reflects the level of consumers’ attention, long-term interest, and attachment to products. In online context, involvement emphasizes the online behavior that forms customer brand and product relationship through cognition, affect, and motivation (Scheinbaum, 2016). The definition of personal involvement used in our study emphasizes an individual’s subjective arousal state (or attention level), or a consumer’s temporary concern about a product shown in the online advertising. Personal involvement can reflect a consumer’s investment in the advertised information and his or her attention upon exposure to advertising (Kim et al., 2018). It can also reflect the level of attention that an individual invests in an item with potential value, which is related to both the perceived value of the item and the relative level of attention (Sheeraz et al., 2018). The study on interactive music by Hwang et al. (2020) has demonstrated that involvement moderates the relationship between interactive music and purchase intention.

In general, consumers hold different attitudes toward differently expressed advertisements (Verhagen and Bloemers, 2018). For instance, they may engage either their “objective rationality” or “subjective sensibility” when choosing between different types of products. Studies have shown that involvement will affect consumers’ information processing, attitudes toward advertising, and decisions to purchase products (Fleury et al., 2016; Harmeling et al., 2017). We propose that when receiving advertised information about a product, a consumer with high personal involvement will invest greatly in and pay much attention to the advertised information. This process stimulates the consumer’s higher internal demand, increasing their desire to further explore the product and the advertised information (Salim and Praningrum, 2018). At this point, consumers will make effective imagination and discover the association between the product and the advertised information. Thus, the matching effects of product type with the expression of the advertised information will be strengthened and help reduce the difficulty that the consumer encounters during information processing, further improving their purchase intention. On the contrary, if the consumer has a low level of personal involvement, they will form an insufficient cognitive memory of the advertised information. At this point, matching the product type to the expression of the advertised information requires the consumer to invest greater effort in understanding the advertised information. This increases the difficulty of information processing and therefore reduces the consumer’s purchase intention. Specifically, we propose the following hypothesis:

*H3*: The consumer’s level of personal involvement positively moderates the effect of matching the product type with the advertising expression on consumer purchase intention.

## Overview of Studies

To test our research hypotheses, we designed three studies based on anchoring theory and the elaboration likelihood model. In the first study, we designed two pilot experiments to identify suitable products and experimental materials. Then, an eye movement experiment was used to prove that the search product advertised with direct expression advertising and the experience product advertised with metaphorical advertising better promoted the purchase intention of consumers. We then further examined the mediate path in the process of the combinative effects of product type and advertising expression on consumers’ purchase intention. In Study 2, we used situational experiments to demonstrate that: (1) matching search products with direct expression advertising and experience products with metaphorical advertising can improve consumers’ purchase intention; and (2) the fluency of information processing is the mediator between matching combination and consumers’ purchase intention mentioned above. In addition, when consumers face a variety of product advertisements, they experience different states of arousal due to different contexts, such as personal characteristic. Therefore, in Study 3, we introduced the boundary condition of consumer personal involvement and further verified its moderating effect.

### Study 1

To select stimuli that would meet the requirements of our eye movement experiment, we conducted two pilot experiments (or “pretests”). Pretest 1 was designed to select two groups of representative product types that were needed for the experiment. Pretest 2 was designed to select the content of the advertised information that was used for each product.

#### Pretest 1: Selection of Representative Products

To select two kinds of products that would be reasonably representative of search and experience products during the experiment, we conducted Pretest 1 in three steps as follows. In the first step, we developed a questionnaire using items from the classification scale for product types by Alok et al. (2004). After extended consideration, we selected 10 kinds of products (e.g., beer, mobile phone, facial masks, and USB flash drives) that had a strong influence on people’s daily lives. In the second step, we randomly recruited 51 undergraduates (with a gender ratio of 9:8) from a university as participants for the pretest. During the pretest, we introduced the participants to the concept of search products and experience products. To test the participants’ understanding of the concept, we then asked them to answer randomly chosen questions about the concept. Finally, we explained the precise concept of product types to the participants before asking them to classify the types of products encountered in the questionnaire. The classifications were made using a 7-level scale ranging from “absolute experience products” (Level 1) to “absolute search products” (Level 7). In the third step, we made a mean analysis of the pretest data for different product types. We found that beer (*M* = 2.86, SD = 1.25) and facial masks (*M* = 2.67, SD = 1.37) had the lowest average scores and thus selected these as representative experience products. We found that mobile phones (*M* = 5.29, SD = 1.06) and USB flash drives (*M* = 5.41, SD = 1.36) had the highest average scores and selected these as representative search products.

#### Pretest 2: Determining Advertised Information for Products

To design the advertised information about the four representative products selected in the Pretest 1, we conducted a second pretest (Pretest 2) in which we compared more than 100 real advertising materials. As all the participants in our formal experiment were expected to be Chinese students, to avoid biases in cognition and attitudes caused by the use of Chinese characters as brand names, we stipulated the following virtual brands of mobile phones, USB flash drives, beer, and facial masks: “DENWA,” “DEISUKU,” “BIIRU,” and “FUAISUPAKU,” respectively. Subsequently, to avoid the possible influence of consumer factors, such as consumers’ familiarity with some real advertisements, specific attitudes toward advertising, and brand impressions resulted from the aforementioned familiarity on the experimental results, we used Photoshop 8.0 software to adjust the size, pixels, word number, and proportion of all images used in the advertised information corresponding to each product. Thus, we used four kinds of products (facial masks, beer, USB flash drives, and mobile phones) and sixteen advertisements (i.e., eight metaphorical advertisements and eight direct advertisements). We showed the sixteen advertisements to fifty-nine undergraduate students from a university in Tianjin. After viewing each advertisement, the participants were asked to immediately rate the extent to which the advertised information reflected a metaphorical statement or a direct statement, the rating was performed using a 7-point Likert scale (i.e., 1 = “complete metaphor” and 7 = “complete literal statement”). An ANOVA (*M*_direct_ = 5.57, SD = 1.06; *M*_metaphor_ = 2.65, SD = 1.25, *F*(1,470) = 746.469, *p* < 0.001) indicated that the manipulation of the stimulus materials met the requirements of our formal experiment.

### Formal Experiment

#### Experiment Design

To identify the best-matched combinations of product types and expressions of advertised information, we tested the effects of the combinations on consumers’ purchase intention in an experiment. Here, we adopted a 2 (product type: search products vs. experience products) × 2 (expression of advertised information: direct vs. metaphorical) two-factor group design. The experimental instrument was a Swedish Tobiix50 eye tracker with a sampling rate of 50 Hz and an accuracy of 0.5°.

The indicators of the experiment were selected following the research of Yang et al. (2018). We selected eye movement indicators, such as fixation times, and total fixation time to measure the participants’ instant state throughout the experiment. The stimuli showed two images—a USB flash drive and a bottle of beer. The images were of the same size and contained the same numbers of pixels and words as well as the same proportion of product shown in each image. In addition, to avoid the “order effect,” which is caused by the left–right order of stimuli presented during eye movement experiments, we used a Latin square (i.e., a random transposition) to solve the problem. We initially recruited 65 graduate students and three participants were deleted as the unsatisfied reliability. Finally, 62 graduate students were as the participants for the study (34 men and 28 women). All of the participants had corrected visual acuity above 1.5, and none had dyslexia.

#### Procedure

To avoid possible interference from sound and light, achieving effective insulation from sound and uniformity in lighting, the entire experiment was carried out in a specialized laboratory used for eye movement tracking. First, we invited each of the 62 participants to enter the laboratory separately and asked them to sit in front of the instrument, allowing the participant to calibrate the eye tracker. Second, following calibration, each participant read the instructions of the experiment, which were displayed on the eye tracker. The eye tracker then automatically displayed the next group of pictures in 30-s intervals. Here, the participants were presented with two groups of unrelated pictures as an exercise task. Finally, the participants began the formal experiments. To reduce potential bias arising from the order effect, we used a Latin square (i.e., a random transposition) to present the images. The experiment lasted approximately 3 min for each participant. At the end of the experiment, we asked each participant to complete a questionnaire based on the product attractiveness scale (Gurhan-Canli and Maheswaran, 2000), using a 7-point Likert scoring method. To ensure that there had been no interference from any participant’s experience of a similar experiment, we included the item “I have completed this type of questionnaire before” in the questionnaire.

#### Manipulation Check and Data Analysis

All data from the experiment were analyzed using Tobii Studio software for eye movement analysis and SPSS 17.0. Prior to conducting statistical analyses, we first determined the areas of interest (AOI). The two AOI in our experiment were set as the two text advertising regions of the same product advertising picture.

An independent-samples *t*-test of the participants’ fixation times revealed a significant difference between the two types of advertised information for search products (*M*_direct_ = 37.65 > *M*_metaphor_ = 19.16, *t* = 7.39, *df* = 50.08, *p* < 0.001), such that the information in direct expression advertising was browsed more times. We also found a significant difference in fixation times between the two kinds of advertised information for experience products (*M*_direct_ = 18.84 < *M*_metaphor_ = 35.32, *t* = 7.64, *df* = 47.59, *p* < 0.001), such that the participants browsed the metaphorical advertisements more times than the direct expression advertisements. The results of an independent-samples *t*-test for total fixation time showed a significant difference between the two types of advertised information for search products (*M*_direct_ = 17.84 > *M*_metaphor_ = 11.39, *t* = 5.64, df = 59.89, *p* < 0.001), such that the total fixation time was longer when direct expression advertisements were used. For the experience product, there was a significant difference in the participants’ total fixation time for the two types of advertised information (*M*_direct_ = 10.47 < *M*_metaphor_ = 18.77, *t* = 9.46, df = 59.79, *p* < 0.001), which suggested that the total fixation time was longer when metaphorical information was used.

After completing the eye movement experiment, we analyzed the data from the questionnaire surveying the participants’ views on the attractiveness of advertising. The results showed a significant difference in the attractiveness of the two kinds of advertised information for search products (*M*_direct_ = 5.26 > *M*_metaphor_ = 2.84, *t* = 8.81, *df* = 59.526, *p* < 0.01). Hence, direct expression advertising of search products was more attractive to consumers than metaphorical advertising. We also found a significant difference in the attractiveness of the two kinds of advertised information for experience products (*M*_direct_ = 3.55 < *M*_metaphor_ = 5.16, *t* = 5.72, *df* = 53.62, *p* < 0.001), suggesting that in this context, metaphorical advertisements are more attractive to consumers than direct expression advertisements.

#### Discussion

By combining an eye movement experiment with statistical analyses of survey data, we empirically analyzed the effect of matching the product type with the expression of advertised information on consumers’ purchase intention. The results from analyses of the participants’ fixation time and total fixation time indicate that consumers pay more attention to direct information in advertisements for search products (vs. experience products) and take more time to contemplate metaphorical information in advertisements for experience products (vs. search products). Overall, the results of our empirical study on consumers’ purchase intention show that direct expression of advertised information is better matched with search products, while, metaphorical expression is better matched with experience products. Such matching combination can help to improve consumers’ purchase intention.

### Study 2

#### Experiment Design

In Study 2, we adopted a 2 (product type: search product vs. experience product) × 2 (expression of the advertised information: metaphorical vs. direct) two-factor group design. We selected the mobile phone, which had a higher mean value in Pretest 1 (*M* = 5.29, SD = 1.064), as the search product, and the facial mask, which had a lower mean value (*M* = 2.67, SD = 1.37) in Pretest 1, as the experience product. We controlled for the color of each product and the participants’ familiarity with the brands. We then simulated an online shopping situation to improve the external validity of the study.

#### Procedure

At the beginning of the study, we first explained the background of the project to all of the participants and informed them that they were participating in a simulated shopping experiment. We used a projector to display pictures of unrelated advertising material to the participants. Then, we asked the participants various questions at random to ensure that they had a clear understanding of the experimental variables. We then asked the participants to rate the product types and the expression of the advertised information displayed in the experimental materials, using the same methods as described for Study 1. Next, based on their specific 2 × 2 two-factor group design mentioned above, the participants were shown images of advertisements for the products. They were allowed to observe each image for approximately 1 min. After the experiment, the participants were required to complete the Information-processing Fluency Assessment Scale (Landwehr et al., 2011; Cronbach’s alpha = 0.917) and the Purchase Intention Scale (Gurhan-Canli and Maheswaran, 2000). Each participant took approximately 5 min to complete the formal experiment. The seven-point Likert scoring method was used. To ensure that there had been no interference from any participant’s experience of a similar experiment, we included the item “I have completed this type of questionnaire before” in the questionnaire.

#### Manipulation Check and Data Analysis

After excluding invalid responses, we collected 400 questionnaires with valid responses (53.8% *Male*; 46.3% *Female*). All participants had corrected visual acuity above 1.5, and none had dyslexia. We conducted a manipulation test on the expression of advertised information and found that the score for metaphorical expression was significantly lower than that for direct expression (*M_metaphor_* = 2.90, SD = 1.40; *M_direct_* = 5.11, SD = 1.44; *F*(1,396) = 243.97, *p* < 0.001). The results confirmed that the manipulation of the expression of advertised information had been successful. We conducted a manipulation test on product type and found that the score for search products was significantly higher than that for experience products (*M_search_* = 3.05, *SD* = 1.44; *M_experience_* = 5.26, SD = 1.25; *F*(1,396) = 269.76, *p* < 0.001). These results confirmed that the manipulation of product category had been successful.

**Figure 1:**
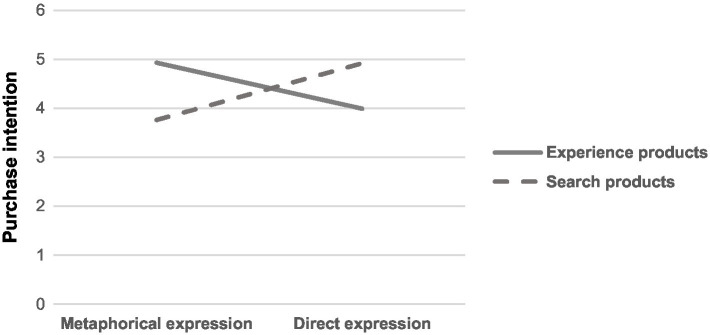
The combined effect of product types and expressions of advertised information on consumers’ purchase intention.

The test for purchase intention revealed that the main effect of product type was not significant [*F*(1,396) = 0.83, *p* > 0.050]; and that the main effect of the expression of advertised information was not significant [*F*(1,396) = 0.412, *p* > 0.050]. However, the interaction effect between the expression of advertised information and product type was significant [*F*(1,396) = 51.72, *p* < 0.001], as shown in Figure 1. We found that for experience products, consumers’ purchase intention was significantly greater in response to metaphorical expressions of advertised information than to direct expressions (*M_metaphor_* = 4.93, SD = 1.48; *M_direct_* = 3.99, SD = 1.38), and this difference was significant [*F*(1,396) = 23.01, *p* < 0.001]. For search products, consumers’ purchase intention was significantly greater in response to direct expressions of advertised information than to metaphorical expressions (*M*_*metaphor*_ = 3.76, SD = 1.60; *M_direct_* = 4.92, SD = 1.46), and this difference was also significant [*F*(1,396) = 28.74, *p* < 0.001]. Taken together, the above results provide converging evidence in support of H1.

Our test of consumers’ information-processing fluency showed that the main effect of product type was not significant [*F*(1,396) = 1.36, *p* > 0.050]; the main effect of the expression of advertised information also was not significant [*F*(1,396) = 0.138, *p* > 0.050]. However, the interaction effect between the expression of advertised information and product type was significant [*F*(1,396) = 55.69, *p* < 0.001]. A further analysis of simple effects showed that for experience products, the consumers’ information-processing fluency in response to metaphorical expressions of advertised information was significantly greater than that in response to direct expressions (*M_metaphor_* = 4.95, SD = 1.27; *M_direct_* = 4.03, SD = 1.21), and this difference was significant [*F*(1,396) = 27.72, *p* < 0.001]. For search products, consumers’ information-processing fluency in response to direct expressions of advertised information was significantly greater than that in response to metaphorical expressions (*M_metaphor_* = 3.83, SD = 1.33; *M_direct_* = 4.84, SD = 1.39), and this difference was significant [*F*(1,396) = 28.08, *p* < 0.001].

Given our focus on matching the expression of advertised information with the product type, it was necessary to verify the role of consumers’ information-processing fluency in shaping the effect of such matching on consumers’ purchase intention. Drawing from the method proposed by Zhao et al. (2010), we used bootstrapping to test the mediating effect of information-processing fluency. We used the process plugin of SPSS, selected model 8 with a sample size of 5,000, and assigned a 95% confidence interval. We found that in the path of “expression of advertised information × product type → information-processing fluency → purchase intention,” the confidence interval for the mediating effect of information-processing fluency is [0.499, 0.739]. The confidence interval does not contain 0, indicating that the mediating effect is significant, with a value of 0.614. Specifically, for experience and research products, in the path of “expression of advertised information → information-processing fluency → purchase intention,” the confidence interval for the mediating effect of information-processing fluency is [−0.2107, −0.0204] and [0.4301, 0.5735] separately, both of which does not contain 0.

Taken together, the results of this study provide converging evidence in support of H2, that is, the fluency of information processing mediates any effect that matching the product type with the expression of advertised information may have on consumers’ purchase intention.

#### Discussion

This study provides an extended empirical analysis on the effect of matching the product type with the expression of advertised information on consumers’ purchase intention and identifies the mediating effect of consumer information-processing fluency on this relationship. The results show that in advertisements for search products, a more direct expression of the advertised information can effectively improve consumers’ purchase intention. For advertisements of experience products, the metaphorical presentation of product information can stimulate consumers’ imagination, thereby enhancing their purchase intention. Finally, consumer information-processing fluency plays a mediating role in the effect of the aforementioned matching of product type and information expression on consumers’ purchase intention.

### Study 3

#### Experiment Design

In this study, we used a 2 (product type: search product vs. experience product) × 2 (expression of advertised information: direct vs. metaphor) × 2 (personal involvement: high vs. low) between-group design, with consumers’ purchase intention assigned as the dependent variable. A total of 731 Chinese consumers were recruited for the experiment. Finally, 720 questionnaires are completed and usable. In the sample, 46.9% were men and 53.1% were women.

#### Procedure

First, we manipulated the consumers’ personal involvement by modifying their situational arousal according to previous research (Barcelos et al., 2018). In the scenario designed to induce low personal involvement, we asked the participants to imagine that they were searching for a mobile phone or facial mask for personal use. In the scenario designed to induce high personal involvement, we asked the participants to imagine that they were searching for a mobile phone or facial mask as a birthday present for a friend who especially likes the brand of the respective product. We then introduced subsequent experimental manipulations following those used in Study 2. At the end of the experiment, we asked the participants to evaluate the expression of advertised information and product types (as was done in Study 1 and Study 2) and to complete the Personal Involvement Scale Cronbach’s alpha = 0.942 and the Purchase Intention Scale (Gurhan-Canli and Maheswaran, 2000).

### Results

#### Manipulation Check and Data Analysis

The manipulation test on the expression of advertised information shows that the score for metaphorical expression was significantly lower than that for direct expression, *M_metaphor_* = 2.94, SD = 1.32, *M_direct_* = 5.11, SD = 1.27, *F*(1,712) = 504.73, *p* < 0.001. The results confirm the successful manipulation of the expression of advertised information. In manipulation tests for product type, we found that search products received significantly higher scores than experience products*, M(search)* = 5.05, SD = 1.383, *M(experience)* = 2.99, SD = 1.390, *F*(1,712) = 396.736, *p* < 0.001. The results confirm the successful manipulation of product type. Furthermore, in a manipulation test, the score for high personal involvement was significantly higher than that for low personal involvement, *M(high)* = 5.05, SD = 0.919, *M(low) =* 3.18, SD = 1.118*, F*(1,712) = 599.904, *p* < 0.001. These results confirm the successful manipulation of personal involvement.

**Figure 2:**
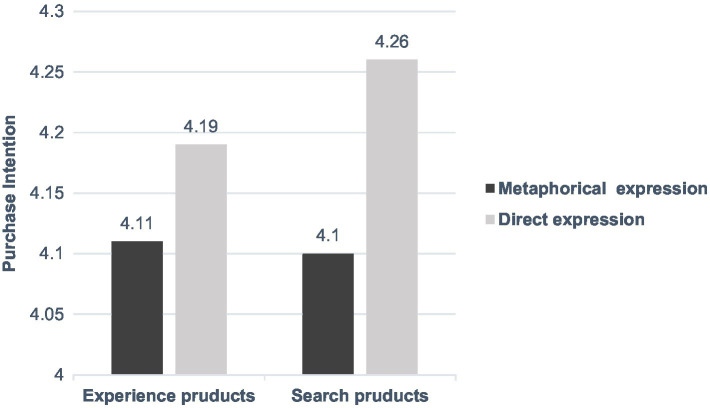
The combined effect of product types and expressions of advertised information on consumers’ purchase intention in low level of personal involvement.

**Figure 3:**
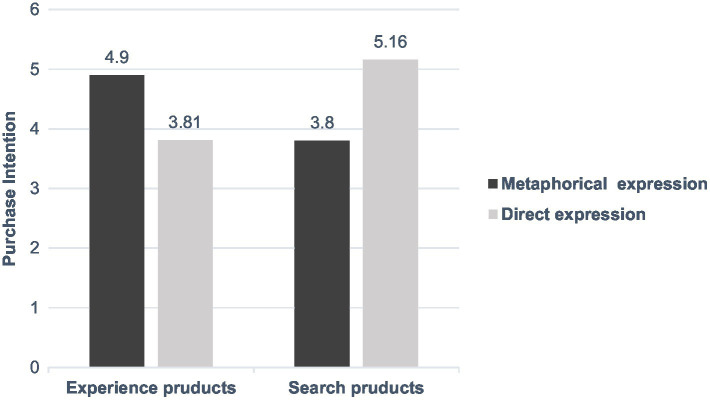
The combined effect of product types and expressions of advertised information on consumers’ purchase intention in high level of personal involvement.

An analysis of variance (ANOVA) showed that a significant main effect of personal involvement [*F*(1,712) = 6.79, *p* < 0.05] and a significant interaction between the expression of advertised information and product type [*F*(1,712) = 37.20, *p* < 0.001]. When personal involvement was low, the effect of the interaction between the expression of advertised information and product type on consumers’ purchase intention was no longer significant (*p* > 0.05). For both experience products and search products, we found no significant difference in consumers’ purchase intention between the different expressions of advertised information (*p* > 0.05). When personal involvement was high, the effect of the expression of advertised information and product type on consumers’ purchase intention was strengthened [*F*(1,712) = 68.76, *p* < 0.001]. For experience products, consumers’ purchase intention in response to metaphorical expressions of advertised information (*M* = 4.90) was significantly higher than those in response to direct expressions (*M* = 3.81). For search products, consumers’ purchase intention in response to metaphorical expressions of advertised information (*M* = 3.80) was significantly higher than those in response to direct expressions (*M* = 5.16). Taken together, the results of Study 3 (shown in Figures 2, 3) provide converging evidence in support of H3.

#### Discussion

The results from this experiment provide empirical evidence of the moderating role of consumer personal involvement in the combined effect of the product type and the expression of advertised information on consumers’ purchase intention. When consumers have a high level of personal involvement, their intrinsic arousal in response to the advertised information is also high, and the positive effect of the relationship between the product type and the expression of advertised information on their purchase intention is strengthened. In contrast, when consumers have a low level of personal involvement, they experience low intrinsic arousal in response to the product type and the expression of advertised information. Thus, any positive effect of the relationship between product type and the expression of advertised information on their purchase intention is weakened.

## Discussion and Conclusion

Drawing on the elaboration likelihood model, anchoring theory, and media richness theory, this study explored the interactive effect of matching the product type with the expression of advertised information on consumers’ purchase intention, and comprehensively analyzed the mediating effect of information-processing fluency and the moderating effect of consumers’ personal involvement on this relationship.

In Study 1, an eye movement experiment was used to demonstrate that the use of more direct expressions of information in advertisements for search products can significantly improve consumers’ purchase intention. In advertisements for experience products, the use of more metaphorical expressions of information can significantly improve consumers’ purchase intention.

In an experiment involving a virtual shopping scenario, Study 2 confirmed that search products are better advertised with direct expressions of advertised information, whereas experience products are better advertised with metaphorical expressions of advertised information. Furthermore, the study empirically demonstrated that these combinations of product type and expression type can significantly enhance consumers’ purchase intention. This study also showed that consumer information-processing fluency mediates the effects of the aforementioned matches between product type and expressions type on consumers’ purchase intention.

Study 3 explored the moderating role of consumer personal involvement in the combined effect of product type and information expression on consumers’ purchase intention. The results from this study show that a consumer with a higher level of personal involvement is more strongly affected by the matching of product type to the expression of advertised information, as a result, their purchase intention increases. However, when a consumer’s level of personal involvement is low, the above effect is weakened.

### Theoretical Implications

Previous studies have mostly explored the effects of singular expressions of advertised information on consumers’ purchase intention (Van Enschot and Hoeken, 2015; Kim et al., 2019). However, with the rise of the Internet and online shopping, in digital context, consumers’ varying aesthetic needs must be met by expressing advertised information in various ways to communicate different product information and to acquire competitive advantage of product rapidly (Amaldoss and He, 2016; Dens et al., 2018; Lee et al., 2019). Few studies have considered the interaction between product type and the expression of advertised information. Our study draws on anchoring theory and media richness theory to develop an integrative theoretical framework to investigate the matching effect of the expressions of advertised information (i.e., direct expression and metaphorical expression) and product types (i.e., search product and experience product) on consumers’ purchase intention. Additionally, although previous studies have considered the direct influence of product type or the expression of advertised information on consumers’ purchase intention, none have examined the possible “black box,” such as the mediating path. In this paper, the “black box” is illuminated from the perspective of information theory, thus, the mediating effect of consumer information-processing fluency is demonstrated. The direct and indirect effects of product type and the expression of advertised information on consumers’ purchase intention are analyzed comprehensively. Finally, although studies have discussed the moderating effects of content consistency (Holmes, 2021) and other external factors on the ways that advertised information shapes consumers’ attitudes, few studies have considered the contingency effects of consumers’ individual characteristics, especially in the matching context. Previous studies also have suggested that personal involvement had crucial attribute for online retailing and marketing research (Leckie et al., 2016; Harmeling et al., 2017). Thus, in this study, consumers’ personal involvement was adopted as a parameter for exploring the boundary conditions on the effects of the main effects. Therefore, our study expands research on the design of product advertising, provides new research perspectives, advances the understanding of product marketing, and enriches the body of knowledge related to anchoring theory and media richness theory.

### Managerial Implications

From the perspective of marketing practice, this study provides useful guidance for enterprises in designing advertisements for different types of products to improve consumer attitudes. First, in digital context, on the one hand, the big data increase the speed of information transfer; on the other hand, the big data increase the competitive intensity of product. Thus, enterprises should emphasize and make full use of product advertising to enhance the effectiveness and efficiency of communication channel to enhance their products’ attractiveness. Based on the findings of our study, we propose that (1) during the early stages of product advertising, enterprises should make full use of social media by including simple and easy-to-understand advertising information that conforms to the characteristics of the product and follows the logic of consumers’ thinking, so as to attract their interest; and (2) in the next stage, enterprises should pay attention to the unique use of vivid ideas, color, sound, and imagery to arouse consumers’ cognitive resources and internal perception toward the product and brand (Burgers et al., 2015). (3) enterprises should match the product to a suitable expression of advertised information to attract potential new consumers exactly.

Second, enterprises should develop plans for advertising different types of products that fully address the characteristics of the products and can enhance consumers’ information-processing fluency, improve their engagement with the target information, and achieve an optimal match between the product type and advertised information. The above measures can remove obstacles that consumers may encounter during the information-processing process. Specifically, before consumers consume real products, they expect to obtain important information about search products through direct and easy-to-understand advertising expressions and to refine this information through rational thought. This process occurs before the consumer takes the center path to judge whether the product is worth buying. Companies can add more intuitive descriptions of the main product information in their advertisements, thus providing consumers with “quick and accurate” information. For experience products, consumers are more willing to obtain product information through metaphorical expressions with associative attributes; these expressions can increase their interest when they are not consuming the product and subsequently generate perceptual purchase intention. Therefore, companies can include more analogies in advertisements and establish a certain degree of association with other metaphors. Such strategies can allow consumers to improve their subjective judgments about products’ utility, thereby stimulating consumption.

Third, in digital era, the scenarios surrounding consumers are rich and dynamic. Enterprises should effectively perceive the emotional and situational needs of consumers in different circumstances and explore the corresponding marketing value. To reach the goal mentioned above, enterprises should create a deeply involved, participatory, and interactive marketing environment for consumers. Such efforts will increase the level of the individual arousal state, attention, as well as the personal involvement from the correlation, interesting, sense of meaning and other aspects, meeting consumers’ demands and reviving the deep “physiological memory,” completely. In such a context, consumer’s subconscious product demand will be stimulated, the attractiveness of the product to consumers is turned into real purchasing power. As a result, the favor of product and brand will be increased.

### Limitations and Future Research

First, besides consumers’ information-processing fluency, there may be other mediators connecting advertising to consumers. Thus, future studies can conduct multi-level in-depth investigations on the internal mechanisms of the main effects of the study and improve the state of the field.

Second, there may be many other boundary conditions on the matching of product type with the expression of advertised information that were not addressed in the present study. For example, controls on the total volume of products in the market may stimulate consumers to compare the respective benefits of these products. Such reductions in supply and product choice may drive consumers to conform or pursue different consumption choices. In addition, studies on consumers with different personality characteristics may also find results that differ from those of the current research. Future studies should seek to test the observed relationships between product type, expression of advertised information, and consumers’ purchase intention in other populations, such as in Western societies.

## Data Availability Statement

The raw data supporting the conclusions of this article will be made available by the authors, without undue reservation.

## Author Contributions

QY contributed to the framework and design of the study. SL organized the database and performed the statistical analysis. HK wrote the first draft of the manuscript. YL contributed to manuscript revision, read, and approved the submitted version. All authors contributed to the article and approved the submitted version.

## Funding

This study was supported by the Philosophy and Social Science Research Project of Tianjin (TJGL21-002).

## Conflict of Interest

The authors declare that the research was conducted in the absence of any commercial or financial relationships that could be construed as a potential conflict of interest.

## Publisher’s Note

All claims expressed in this article are solely those of the authors and do not necessarily represent those of their affiliated organizations, or those of the publisher, the editors and the reviewers. Any product that may be evaluated in this article, or claim that may be made by its manufacturer, is not guaranteed or endorsed by the publisher.
